# Genetic Diversity, Population Structure and *Wolbachia* Infection Status in a Worldwide Sample of *Drosophila melanogaster* and *D. simulans* Populations

**DOI:** 10.1371/journal.pone.0026318

**Published:** 2011-10-12

**Authors:** Rudi L. Verspoor, Penelope R. Haddrill

**Affiliations:** Institute of Evolutionary Biology, School of Biological Sciences, University of Edinburgh, Edinburgh, United Kingdom; University of Cambridge, United Kingdom

## Abstract

*Drosophila melanogaster* and its close relatives have been extremely important model species in the development of population genetic models that serve to explain patterns of diversity in natural populations, a major goal of evolutionary biology. A detailed picture of the evolutionary history of these species is beginning to emerge, as the relative importance of forces including demographic changes and natural selection is established. A continuing aim is to characterise levels of genetic diversity in a large number of populations of these species, covering a wide geographic area. We have used collections from five previously un-sampled wild populations of *D. melanogaster* and two of *D. simulans*, across three continents. We estimated levels of genetic diversity within, and divergence between, these populations, and looked for evidence of genetic structure both between ancestral and derived populations, and amongst derived populations. We also investigated the prevalence of infection with the bacterial endosymbiont *Wolbachia*. We found that *D. melanogaster* populations from Sub-Saharan Africa are the most diverse, and that divergence is highest between these and non-Sub-Saharan populations. There is strong evidence for structuring of populations between Sub-Saharan Africa and the rest of the world, and some evidence for weak structure amongst derived populations. Populations from Sub-Saharan Africa also differ in the prevalence of *Wolbachia* infection, with very low levels of infection compared to populations from the rest of the world.

## Introduction

Understanding the forces that shape patterns of genetic diversity within and between population and species is a major goal of evolutionary biology. Contemporary patterns of genetic variability result from a combination of evolutionary processes in the history of a population or species, including demographic size changes and the action of natural selection [1]–[3]. Determining the relative importance of these forces in different populations is essential if we are to understand how species have evolved, and this requires a detailed knowledge of how genetic diversity is distributed around the genome and within and between both populations and species.

*Drosophila melanogaster* and its close relatives have been important model species in the development of population genetic models that serve to explain patterns of genetic diversity in natural populations [4], [5]. Levels of variability have been characterised in numerous *D. melanogaster* populations from around the species range, and models that include the effects of population size changes and the impact of both positive and negative selection have been developed to attempt to explain patterns of diversity and to determine the evolutionary history of populations (e.g. [6]–[9]). These studies consistently show that populations found in Sub-Saharan Africa have much higher levels of genetic diversity than those outside Africa, and suggest an East African origin for the species [10]–[19]. Demographic size changes have been shown to be important in the history of the species, with bottlenecks occurring during colonisation of habitats outside Africa [10], [16], [17], [20], [21], and there is also evidence that African populations have experienced increases in population size [13], [22],[23]. Selective forces are also likely to have been important in the history of the species, particularly as derived populations adapt to new habitats in temperate regions [13], [24]–[27]. The timing of this expansion around the world has also been estimated, with the spread from the ancestral range in Africa into Europe thought to have occurred around 16,000 years ago [16], [17], [20]–[22], and into North America only a few hundred years ago [4], [5].

A more detailed picture is thus emerging of the evolutionary history of this species, and some of its close relatives [10], [28]–[34]. However, many of these studies have focused on the same few populations as representative examples of ancestral and derived populations (e.g. [13], [17], [21]–[23]). In order to get a clear and detailed picture of the distribution of genetic diversity for a species, it is essential to study a large number of populations, covering a wide geographical area. In addition, most studies have used isofemale lines that have been inbred in the laboratory for many generations. Although adjustments are made for the resulting loss in variability when calculating diversity statistics, this does not directly measure the levels of diversity in wild populations, and chance founder effects within isofemale lines could inflate estimates of divergence between populations.

Finally, in order to understand all of the forces that are important in shaping patterns of diversity in wild populations, it is also important to consider the impact of ecological variables. Differences in the variance in reproductive success between the sexes and between populations and species have impacts on the distribution of genetic diversity around the genome and among populations [35]–[38], and there is evidence to suggest that these forces may be important in *D. melanogaster* and *D. simulans* [39]–[43]. The presence of cytoplasmically inherited endosymbionts may also be relevant in this context, particularly if they distort population sex ratios, and they may have important implications for both natural and sexual selection and thus for patterns of molecular evolution [44]. To understand the importance of these effects, a key aim is to begin characterising a number of ecological variables in specific wild populations from around the species range. To complement this, we therefore need detailed genetic diversity data from the same specific populations.

We have characterised genetic diversity in, and divergence between, five newly sampled wild *D. melanogaster* and two *D. simulans* populations, collected from three continents. We also look for evidence of structuring of populations both between ancestral and derived populations, and amongst derived populations. In addition, we investigate the prevalence of infection with the bacterial endosymbiont *Wolbachia*. We find patterns of genetic diversity and divergence that are in general agreement with previous reports for other populations in these species, and also document striking differences in the rate of *Wolbachia* infection between populations.

## Materials and Methods

### Population Samples

Five populations of *Drosophila melanogaster* were included, from the following locations: Athens in Georgia, USA, collected by P. Haddrill in August 2009; Accra in Ghana, West Africa, collected by P. Haddrill in January 2010; Montpellier in France, collected by P. Haddrill in August 2010; Marrakech in Morocco, North Africa, collected by C. Webster in September 2010 and Sussex in the UK, collected by D. Obbard in July 2011. We also included a population of *D. simulans* from Athens in Georgia, USA, collected by P. Haddrill in August 2009. For each population, multiple isofemale lines were allowed to establish in the lab for a maximum of five generations before a single female was sampled from each, except for the Sussex population, where individuals were sampled from the F1 generation. For four of the populations, we also added a number of single females sampled directly from the wild. The final sample sizes for each of the *D. melanogaster* populations were therefore 57 for Georgia (32 lines and 25 additional females), 101 for Ghana (76 lines and 25 additional females), 63 for France (39 lines and 24 additional females), 18 for Morocco (isofemale lines only) and 38 for Sussex (isofemale lines only). The *D. simulans* sample from Georgia consisted of 19 lines and 22 additional females, giving a final sample size of 41. We also included a single female from each of five *D. simulans* isofemale lines from the French population, which were originally identified as *D. melanogaster*. Although the sample size for this population is small and thus estimates of diversity and divergence should be treated with caution, five individuals provide information about ten wild alleles, and were therefore considered worth analysing. Species identification for each of the 323 females included in the study was confirmed using a species-specific PCR.

### Screening for Wolbachia

All the females included in the study were assayed for the presence of *Wolbachia* infection using PCR primers to amplify part of the *wsp* gene [45]. Reactions were carried out in a volume of 25 µL, containing 40ng of genomic DNA as template, 2.5 µL of 10x PCR buffer (containing 15mM MgCl_2_), 0.5 µL of dNTPs (10mM), 1 µL of each primer (10 µM) and 0.1 µL of *Taq* polymerase (5 units/ µL, Roche Diagnostics). Cycling conditions on a G-Storm thermal cycler were 94°C for 5 minutes followed by 35 cycles of 94°C for 30 seconds, 55°C for 45 seconds and 72°C for 1 minute, with a final extension step of 72°C for 5 minutes. Amplification for all samples, and for known positive and negative control samples, were checked by running on a 1% agarose gel. A subset of individuals were tested twice to confirm their infection status.

### Microsatellite genotyping

All females were genotyped for nine microsatellite loci from around the *D. melanogaster* genome, of which three were X-linked and six were autosomal (two on the second chromosome and four on the third chromosome). These loci were a subset of those developed by Orozco-terWengel et al [46] and were multiplexed into a single panel of loci for genotyping using fluorescently labelled forward primers. The loci and the fluorescent dye used for each (in parentheses) were as follows: 2r3m4 (VIC), 2r5m2 (6FAM), 3r1M5New (6FAM), 3r2M8 (PET), 3r4M2 (6FAM), 3r5M7 (VIC), XR1M8 (PET), Xr4M10 (6FAM) and Xr4M5 (VIC). PCR reactions were carried out in a volume of 20 µL containing 100ng of genomic DNA as a template, 6.4 µL of 5x PCR buffer, 2.0 µL of MgCl_2_ (25mM), 0.4 µL of dNTPs (10mM), 0.2 µL of each primer (20 µM) and 0.4 µL of hot start *Taq* polymerase (5 units/ µL, Promega Corporation). PCR products were analysed on an ABI3730 DNA Analyzer (Applied Biosystems) and alleles were scored using GeneMapper software version 4.0 (Applied Biosystems).

### Analysis

Several measures of population diversity and divergence were calculated using Microsatellite Analyser (*MSA*), version 4.05 [47]. The expected heterozygosity (*H*_E_), or gene diversity, was calculated both using the full dataset and by taking the average of 200 resampled datasets in which one of the two alleles at each locus is randomly discarded. This was done in order to examine whether there had been any impact on genetic diversity of the five generations of inbreeding in the samples that came from isofemale lines. Allelic richness was estimated using the minimum number of individuals successfully genotyped per locus [48], [49]. *MSA* was also used to calculate pairwise estimates of *F*_ST_ [50] between populations, and the statistical significance of these values was tested by permutation of genotypes 10,000 times among populations; *P*-values were adjusted for multiple testing using a Bonferroni correction [51].

Population structure was assessed using a Bayesian model-based approach, implemented in the program *Structure*, version 2.3, which detects genetic clusters within the entire sample without using information regarding the sampling location of each individual, and then assigns proportions of the ancestry of each individual into the different clusters [52]. We analysed the data under the admixture model, with correlated allele frequencies, since we expect all populations to have shared ancestry [53]. Using a burn-in length of 1×10^5^ and a run length of 1×10^6^, we carried out ten independent runs of the model for each of *K* (the number of populations)  = 1 to *K* = 8. In order to assess whether the burn-in and run lengths were sufficient, we checked that key parameters such as *F*_ST_ and alpha (the Dirichlet parameter representing the degree of admixture) converged before the end of the burn-in period, and that the variation in log likelihoods across multiple runs of the program at each *K* value was smaller than the variation between runs at different *K* values. Following the removal of the Ghana population from the analysis (see Results and Discussion section below), we carried out ten independent runs of the model for each of *K* = 1 to *K* = 8 under the LOCPRIOR model, which uses the sampling locations of individuals to assist in the detection of genetic structure when the signal is too weak to be found using the standard models [54]. As previously, we used the admixture model with correlated allele frequencies, and burn-in and run lengths of 1×10^5^ and 1×10^6^, respectively. Ancestry assignments to each cluster for each individual in the sample were visualised using *Distruct* [55].

## Results and Discussion

### Wolbachia infection

Table 1 shows the infection status and prevalence of the bacterial endosymbiont *Wolbachia* in each of the five populations of *D. melanogaster* and in two *D. simulans* samples. The major patterns of note are, firstly, that *Wolbachia* infection is found in all populations and, secondly, that prevalence is high in the majority of them. We tested for differences in prevalence levels between populations using a Fisher's Exact Test for within-species data. Whilst there were no differences in infection level between *D. simulans* populations (*P* = 1.0), there were highly statistically significant differences between *D. melanogaster* populations (*P*<10^−16^). This pattern is not driven entirely by the results for the Ghana population (see below); when this population is removed there is still statistically significant evidence for heterogeneity in infection levels (*P* = 0.026).

**Table 1:** Infection status of females in five populations of *D. melanogaster* and two populations of *D. simulans* for the bacterial endosymbiont *Wolbachia*.

Population	*n*	No. infected	Prevalence
*D. melanogaster*			
USA	57	34	59.65% (45.82 – 72.44%)
Ghana	101	1	0.99% (0.03 – 5.39%)
France	63	30	47.62% (34.88 – 60.59%)
Morocco	18	15	83.33% (58.58 – 96.42%)
UK	38	26	68.42% (51.35 – 82.50%)
*D. simulans*			
USA	41	37	90.24% (76.87 – 97.28%)
France	5	5	100.00% (47.82 – 100.00%)

*n* =  sample size, prevalence given with 95% confidence intervals in parentheses, estimated using the Clopper-Pearson method.

The *D. simulans* sample showed the highest level of infection, with over 90% of individuals from Georgia, USA being infected. In addition, all of the French *D. simulans* were infected, although there were only five individuals in this sample, so this prevalence level should be interpreted with caution. These values are close to the expected equilibrium frequency for the *Wolbachia* strain that infects *D. simulans*, *w*Ri, which is predicted to be around 94% infection [56], [57], and are consistent with infection frequencies in other North American populations of *D. simulans* [58].

Within the *D. melanogaster* sample, *Wolbachia* was highly prevalent in all of the non-Sub-Saharan Africa populations, with around 50–80% of individuals being infected. This is somewhat higher than previous estimates of *Wolbachia* prevalence in *D. melanogaster*, which suggest around 30–45% of stock centre lines are infected [59]–[61]. However, estimates of infection level in wild populations (rather than stock centre lines) from Australia are consistent with our results, with high prevalence in more northerly populations, although this drops rapidly in populations further south [62]. In contrast, we find only a single individual infected with *Wolbachia* in Ghana, suggesting that prevalence is less than 1% in this population. Although estimates from sub-Saharan Africa are relatively scarce, this is in disagreement with prevalence estimates from stock centre lines of African origin [59], [61], and with estimates from a wild Ugandan population [63]. It is also of note that previous studies have consistently found some populations that seem to be completely uninfected, whereas *Wolbachia* is present in all of our sampled populations [59]–[61]. This may reflect the continuing spread of infection around the world or, in the case of the Ghana population at least, the influence of sample size on the probability of detecting infection.

Since infection status has not been assayed in the Ghana population before, it is unclear whether this low level of infection is a result of a prior infection having been lost, or because the population has not previously been infected. Given that *Wolbachia* causes only weak cytoplasmic incompatibility in the wild, the infection is predicted to be lost from populations, unless it also confers a fitness benefit to the host [62], [64]. There is some evidence suggesting positive effects of *Wolbachia* infection on fitness in *D. melanogaster*, although these effects vary depending on a number of factors, including the sex and the genetic background of the host [65]–[67]. The strength of *Wolbachia*-induced cytoplasmic incompatibility also varies with age, and can be much stronger when infected males mate very young, which has also been postulated to explain the persistence of infection in wild populations [68]. If any positive fitness effects of *Wolbachia* infection vary between populations, or if there are differences in mating behaviour, such that males tend to mate at different ages in different populations, this might lead to marked differences in infection level between populations. Differences in the relative levels of genetic diversity found on the X chromosome and autosomes of *D. melanogaster* from African and non-African populations [40]–[43] suggest that there may be differences in mating-related traits between these populations, particularly in terms of the strength of sexual selection on males [35]–[38]. It is possible, therefore, that these differences could affect the dynamics and persistence of *Wolbachia* infection within these populations.

One additional factor that should be considered is that the *D. melanogaster* populations could be infected with different strains of *Wolbachia*, although surveys of long-term lab stocks originating from five continents (including North America, Africa and Europe) suggest that one of these strains has replaced all others over the last ∼50 years [61]. It is possible that differences in prevalence level between populations could correspond to differences in the specific strain infecting the population, but since the *wsp* gene has been shown to be uninformative in distinguishing between different *Wolbachia* strains [69], we are unable to determine strain information here.

### Genetic diversity

We generated microsatellite data for nine loci in 323 individuals from five populations of *D. melanogaster* and two populations of *D. simulans*, a total of almost 3000 genotypes. For comparison, we added data on genetic diversity at the same loci for the Victoria Falls Zimbabwe (Zw) population studied by Orozco-terWengel et al [46]. Table 2 shows several measures of genetic diversity in each of the populations. For each population (apart from Zimbabwe, which consisted of inbred isofemale lines), we calculated the mean expected heterozygosity both from the full dataset and from the mean of 200 datasets generated by randomly discarding one allele at each locus. This was done to examine any effects of the five generations of inbreeding that may have occurred in isofemale lines from some of the populations. In every case there was less than 0.15% difference between the two estimates, so we can conclude that short-term inbreeding in our isofemale lines has not substantially affected measures of genetic diversity.

**Table 2:** Microsatellite diversity statistics within six *D. melanogaster* and two *D. simulans* populations.

Population	*n*	*H*_E_	*H*_E(RD)_	No. of alleles	Allelic richness
*D. melanogaster*					
USA	57	0.42	0.42	5.78	4.14
Ghana	101	0.59	0.59	9.11	5.87
France	63	0.37	0.37	4.78	3.67
Morocco	18	0.30	0.31	3.67	3.67
UK	38	0.35	0.35	4.00	3.46
Zimbabwe	24	-	0.67	5.58	5.92
*D. simulans*					
USA	41	0.42	0.42	3.44	3.14
France	5	0.41	-	2.22	-

*n* =  sample size, *H*_E_  =  mean expected heterozygosity across loci, *H*_E(RD)_  =  mean expected heterozygosity calculated from 200 datasets generated by randomly discarding one allele at each locus, Zimbabwe data taken from [46], *H*_E(RD)_ and allelic richness were not calculated for the France *D. simulans* population, because of the small sample size.

As expected, within our *D. melanogaster* sample, the Ghanaian population consistently shows the highest levels of genetic diversity for all measures, exhibiting a mean expected heterozygosity almost double that of the least diverse population, Morocco. The mean number of alleles per locus and the allelic richness is also markedly higher in Ghana than in all other sampled populations, and whilst this population does have somewhat lower gene diversity than the East African Zimbabwe population, its allelic richness is very similar and the mean number of alleles per locus is higher than in Zimbabwe, probably reflecting the large sample size for the Ghanaian population. This is in general agreement with previous studies showing that West African populations have levels of diversity higher than populations outside Sub-Saharan Africa [15], [18], but somewhat lower than East African populations [14], [16]. Diversity in the Moroccan population is also consistent with previous reports that North African populations harbour lower levels of genetic variability than populations in Sub-Saharan Africa, although these have tended to find higher levels of diversity in North Africa than in European populations ([18], [19], although see [15]), which is contrary to our findings. Nevertheless, despite higher levels of diversity than the North African population, the European populations have substantially reduced levels of diversity relative to the two populations from Sub-Saharan Africa, and also have lower levels of diversity than the North American population, consistent with previous studies [11]–[14], [16], [43].

The North American *D. melanogaster* population has a diversity level intermediate between the European and Sub-Saharan Africa populations, but similar to *D. simulans* from the same location. This is somewhat surprising given that *D. simulans* is generally thought to have a larger effective population size than *D. melanogaster*, based on reports that it has higher levels of genetic diversity [70]–[72], although studies of X-linked loci have reported similar levels of variability between the two species [33], [34], [40]. The French *D. simulans* sample does indeed exhibit higher diversity than the French *D. melanogaster* sample, although this is based on a small sample size for *D. simulans*. It should also be noted that the microsatellite markers used were developed in *D. melanogaster*, and thus were selected on the basis of being polymorphic in that species. They therefore may not be expected to be as polymorphic in another species, although comparison of the expected heterozygosity for individual loci in the two species from the Georgia population shows that *D. melanogaster* exhibits higher diversity at five loci, and *D. simulans* at four (data not shown).

### Genetic differentiation

Estimates of genetic differentiation between populations are shown in Table 3, which contains *F*_ST_ estimates between all pairs of populations. Significant levels of differentiation were found between all pairs of populations except between the two *D. simulans* populations (although the small sample size for the French population should be borne in mind), and between the French and Moroccan *D. melanogaster* populations. We found varying levels of divergence between different populations of *D. melanogaster*; *F*_ST_ estimates are highest between Ghana and the European/North African populations (∼0.24), but are considerably lower between Ghana and the North American population (0.18), suggesting that North American populations are more closely related to ancestral African populations than European and North African populations are. This has been reported previously and is thought to result from continued or more recent admixture from Africa into North American populations [12], [14], [19], [46]. It has also been proposed that North American populations may be subject to admixture with African alleles originating from Central and South American populations, which have been shown to be segregating for African traits [12], and also show lower levels of reproductive isolation from West African populations than from East African and North American populations [73]. It is possible that this is a contributing factor in explaining the relatively low level of genetic differentiation seen between the West African and North American populations.

**Table 3:** Pairwise *F*_ST_ estimates between populations and associated statistical significance (above diagonal), corrected for multiple tests using a Bonferroni correction.

		*D. melanogaster*					*D. simulans*	
		USA	Ghana	France	Morocco	UK	USA	France
*D. melanogaster*	USA	–	0.180**	0.038**	0.042**	0.034**		
	Ghana		–	0.248**	0.234**	0.239**		
	France			–	0.023^NS^	0.033**		
	Morocco				–	0.042**		
	UK					–		
*D. simulans*	USA						–	-0.029^ NS^
	France							–

**p<0.01, NS  =  non-significant.

Our measures of differentiation between Sub-Saharan Africa and European populations of *D. melanogaster* are consistent with several previous estimates of divergence between African and European populations [15], [19], [46], although estimates based only on X-linked data tend to be somewhat higher [11], [14], [18], and some based on autosomal data somewhat lower ([11], see also [12], [19]). The *F*_ST_ estimates between the Moroccan population and the Ghana population are also higher than some previous estimates of population differentiation between Sub-Saharan African and North African populations ([15], [19], although see [18]).

Levels of divergence are fairly similar among all pairs of non-Sub-Saharan Africa populations, although the North American population is somewhat more divergent, and the Moroccan and French population are somewhat more closely related. The *F*_ST_ estimates between these populations range from ∼0.02 – 0.04, which are consistent with, although a little lower than some previous estimates of genetic differentiation between European, North African and North American populations [11], [12], [14], [15], [18], [19], [46]. These slightly lower estimates of population differentiation may reflect the fact that we have used new wild collections, and thus there has been less opportunity for founder effects during the establishment of isofemale lines to influence measures of genetic divergence between samples.

### Population structure

**Figure 1:**
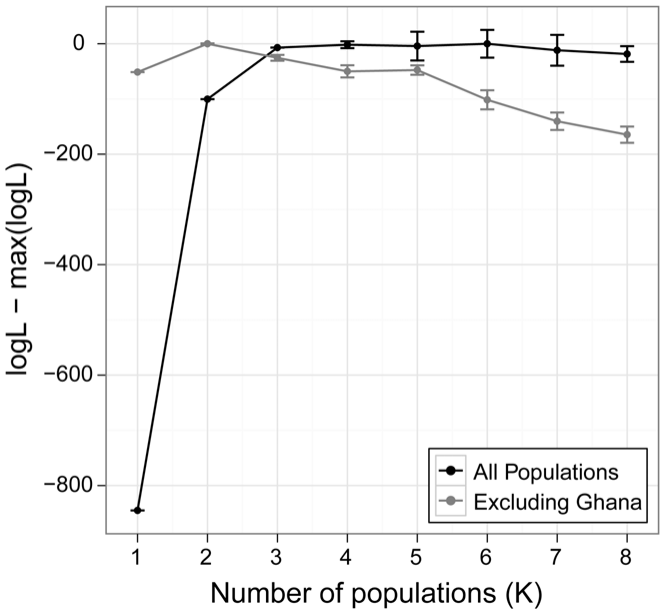
Estimates of the most likely number of populations in a worldwide sample of *D. melanogaster*. The difference between the maximum likelihood and the likelihood for each estimate of *K* (the number of populations) is shown. Two analyses were carried out; one including all populations, and one excluding the population from Ghana.

**Figure 2:**
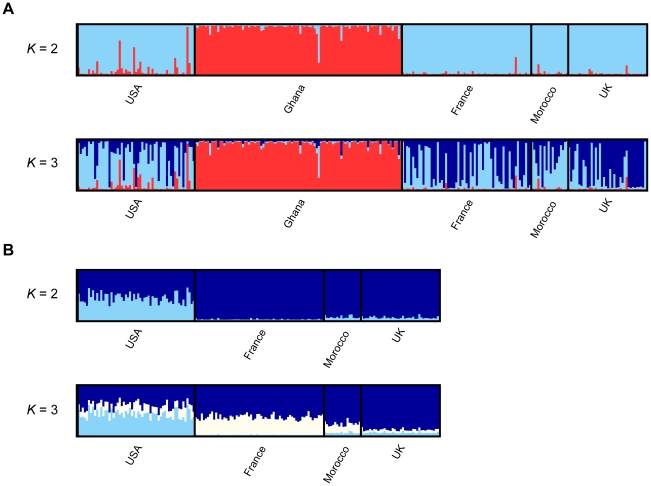
Estimated population structure in a worldwide sample of *D. melanogaster*. The proportion of ancestry for each individual in each of *K* (the number of populations)  = 2 or *K* = 3 clusters is shown. Two analyses were carried out; one including all populations (A), and one excluding the population from Ghana (B).

We investigated these patterns in more detail using a Bayesian approach to detect any genetic structure between the five populations of *D. melanogaster*, implemented in the software *Structure*. We first analysed the data without using information about sampling location, and estimated the likelihood of the data for values of *K* (number of populations) from 1 to 8. Figure 1 shows the mean log likelihood from ten independent runs for each value of *K*. Whilst there is a large increase in log likelihood between the model run under *K* = 1 and *K* = 2, for values of *K* greater than 2, the increase in likelihood is much smaller, particularly when *K* is greater than 3. The *Structure* manual suggests that the best estimate of the true value of *K* is to be found at the point where the likelihoods begin to plateau, suggesting that subdivision of our samples into two or three genetically distinct clusters allows us to capture the major patterns of structure in the data. Figure 2A shows the assignment of all individuals in the sample into two clusters, separated by population, and shows that the major division in the dataset is between the Sub-Saharan Africa population from Ghana and the remaining populations from the rest of the world. This is consistent with previous studies that have found that African and European populations fall into two distinct genetic clusters [11], [16], [18], [46]. Interestingly, the Georgia population seems to have a slightly higher proportion of ancestry in the ‘African’ cluster than the other non-Sub-Saharan Africa populations, which is consistent with the *F*_ST_ results reported above.

We therefore find strong support for the existence of two distinct populations within our data, representing Sub-Saharan Africa and the rest of the world, suggesting that there may be no significant genetic structuring of populations outside Africa. However, the *F*_ST_ results suggest that there is low but significant genetic differentiation between individuals from different locations. It is therefore possible that there is weak structure that the standard models implemented in *Structure* are unable to detect. For example, the results above suggest that there may be differences between the USA and European/North African populations, and some previous studies find that these represent separate populations based on similar Bayesian analyses of population structure ([12], [19], although see [16]). Indeed, the log likelihood does continue to increase for *K*>2 populations, and examining the ancestry of individuals when *K* = 3 (Figure 2A) shows that whilst all the Ghanaian individuals have the majority of their ancestry in one cluster, a large fraction of the remaining individuals in the sample are strongly assigned to one or other of the two non-Ghanaian clusters. The assignments into each of these clusters is also somewhat asymmetric amongst populations, with the European and North African individuals tending to be more strongly assigned to one cluster, and the Georgia individuals being more strongly assigned to the other. This suggests that there might therefore be genuine structuring of non-Sub-Saharan Africa populations, but that it is much weaker than the division between Ghana and the rest of the populations, and thus difficult to detect.

We therefore used the LOCPRIOR model to investigate this further, by using information about the sampling locations of individuals to improve the ability of the program to detect weak population structure. This is a recent development of the program that allows the sampling location of each individual to be included in the model and, in the event that the location is informative in terms of detecting population structure, to make use of this information [54]. We excluded the data for individuals from the Ghana population from this part of the analysis, since we are trying to detect weak structure in the data and the signal of structure from the Ghana population is very strong. Figure 1 shows the log likelihood of the data under different values of *K*, from 1 to 8. As before, the largest difference in log likelihood is between *K* = 1 and *K* = 2, suggesting that there is significant genetic structuring within the non-Sub-Saharan Africa populations. However, the difference between the log likelihood for *K* = 2 and *K* = 3 is again small, so we examined the assignment of ancestry for all individuals into each of *K* = 2 or *K* = 3 clusters (see Figure 2B). When *K* = 2, there is a clear distinction between the population from Georgia, USA and the remaining populations, with ancestry for individuals in the USA being split approximately equally between the two genetic clusters. This may reflect the more recent admixture from African populations into the USA, consistent with the results reported above. In contrast, the European and North African populations have almost all their ancestry in a single cluster, with only very minor differences between them; the Moroccan and UK populations have approximately 5% of their ancestry in the second cluster, whilst individuals from the French population have almost no ancestry assigned to that cluster at all (∼1%).

However, when we examine the ancestry assignments for *K* = 3, we see more pronounced differences between these populations. Although there is not strong statistical support for division of these populations into three clusters, there are some individuals very strongly assigned to some clusters, and the proportion of individuals assigned to each cluster is asymmetric, and these are indications that there may be real population structure. If indeed there are three distinct clusters, one cluster again makes up around 45% of the ancestry of individuals from Georgia, 5% for individuals from Morocco and the UK and 1% for individuals from France. A second cluster makes up around 40% of the ancestry of most French individuals, 15–20% of the ancestry of individuals from Georgia and Morocco, but only about 5% of the ancestry of UK individuals. The final cluster makes up the remaining ∼60% of ancestry of French individuals, 75% of ancestry in Morocco, ∼40% of ancestry in the USA population and approximately 90% of the ancestry in individuals from the UK population. These patterns actually agree reasonably well with the *F*_ST_ results reported earlier, with the Georgia population being the most distinct, little genetic differentiation between France and Morocco, and the UK population falling in the middle. Thus, by using information about the sampling locations of individuals in the sample, we find clear evidence that the population from Georgia is distinct from the remaining non-Sub-Saharan Africa populations, and patterns suggestive of there being further differentiation between the UK population and another group comprising the French and Moroccan populations.

### Summary

We examined genetic diversity, differentiation and population structure amongst five newly sampled wild populations of *D. melanogaster* and two of *D. simulans* spread across Sub-Saharan Africa, North Africa, Europe and North America. We also examined the prevalence of *Wolbachia* infection in each of these samples. We found very high rates of infection with *Wolbachia* in *D. simulans*, consistent both with other estimates of infection prevalence and theoretical predictions of the equilibrium frequency of infection in this species [56]–[58]. In *D. melanogaster*, we find infection levels of 50% or greater in populations from North Africa, Europe and the USA, consistent with some previous estimates from wild populations [62], but higher than estimates based on stock centre lines [59]–[61]. In contrast we find very low levels of *Wolbachia* infection in the Ghana population, contrary to previous estimates from other Sub-Saharan African populations [59], [61], [63]. This could be due to temporal fluctuations in the prevalence of *Wolbachia* infections, which are known to change very rapidly (e.g. [58], [61]), and it is possible that proposed differences in mating behaviour between populations in Sub-Saharan Africa and those outside could have an impact on the population dynamics of *Wolbachia* infection persistence [35]–[37], [40]–[43].

In terms of genetic diversity and differentiation between populations, we find patterns that are generally consistent with previous studies of *D. melanogaster* populations around the world. Using data from an East African population [46], we find that diversity is highest in East Africa, that the West African population has slightly reduced levels of diversity [14], [16], and that all populations outside Sub-Saharan Africa have dramatically reduced levels of diversity [11]–[13], [15], [17]–[19]. Our finding that European and North African populations harbour similar levels of diversity is somewhat at odds with previous studies that have found higher diversity in North Africa ([18], [19], although see [15]), but all three populations in the Europe/North Africa group have lower levels of diversity than the USA population, and this is consistent with other reports [11]–[14], [16], [43]. We also find similar levels of genetic diversity for both *D. melanogaster* and *D. simulans* in the USA population. Although this is contrary to expectations based on reported differences in effective population size between the two species ([70]–[72], although see [33], [34], [40]), this is the first report we are aware of surveying the same set of markers in samples of the two species from the same locality, which may make these more reliable estimates of relative levels of genetic diversity.

Estimates of genetic differentiation also agree well with expectations; within *D. melanogaster*, European and North African populations show the highest level of differentiation from the West African population, whilst estimates are much lower for the USA population. This may reflect more recent admixture of African alleles into the USA, either directly or via Central or South American populations [12], [14], [19].

Finally, we used a Bayesian analysis to detect any genetic structure between the five populations of *D. melanogaster*, an approach that is now widely used to infer population structuring and biogeography amongst multiple populations within species. One important caveat with this analysis is that it has recently been shown that it can result in statistically well-supported but incorrect inferences of population structure, even when large numbers of loci are used [46]. This results from random differences in lineage sorting for different marker loci, and is particularly pronounced for low levels of population differentiation (*F*_ST_<0.1) [46]. This is therefore probably not an issue for comparisons between populations in Sub-Saharan Africa and those outside, but may influence the detection of population structure amongst non-Sub-Saharan Africa population. Thus we should be cautious about drawing firm conclusions based on these results if they are not supported by other aspects of our analyses. Nevertheless, where patterns are consistent both with the *F*_ST_ results and with previous studies, we can be relatively confident in their reliability. In general, our results are consistent with previous studies, in that we find that Sub-Saharan Africa individuals cluster into one population that is distinct from the samples from the rest of the world ([12], [19], although see [16]). We also find that the USA population is somewhat distinct from the European/North African populations, which has also been reported previously [12], [19]. However, previous studies have not examined structuring between populations in Europe/North Africa, whereas recent developments in the *Structure* methods [54] allow us to look for weak genetic structure between these populations. We find that there are indeed some differences between these populations, with the sample from the UK being somewhat distinct from the French and Moroccan populations, all of which is consistent with the *F*_ST_ results. Thus there may be significant genetic structuring of populations outside of Sub-Saharan Africa and amongst populations in Europe.
